# Rapid Assessment of the Potential Paucity and Price Increases for Suggested Medicines and Protection Equipment for COVID-19 Across Developing Countries With a Particular Focus on Africa and the Implications

**DOI:** 10.3389/fphar.2020.588106

**Published:** 2021-01-14

**Authors:** Israel Abebrese Sefah, Olayinka O. Ogunleye, Darius Obeng Essah, Sylvia A. Opanga, Nadia Butt, Annie Wamaitha, Anastasia Nkatha Guantai, Ibrahim Chikowe, Felix Khuluza, Dan Kibuule, Lahya Nambahu, Abdullahi Rabiu Abubakar, Ibrahim Haruna Sani, Zikria Saleem, Aubrey C. Kalungia, Thuy Nguyen Thi Phuong, Mainul Haque, Salequl Islam, Santosh Kumar, Jacqueline Sneddon, Joshua Wamboga, Janney Wale, Nenad Miljković, Amanj Kurdi, Antony P. Martin, Brian Godman

**Affiliations:** ^1^ Pharmacy Department, Keta Municipal Hospital, Ghana Health Service, Keta-Dzelukope, Ghana; ^2^ Pharmacy Practice Department of Pharmacy Practice, School of Pharmacy, University of Health and Allied Sciences, Ho, Ghana; ^3^ Department of Pharmacology, Therapeutics and Toxicology, Lagos State University College of Medicine, Ikeja, Nigeria; ^4^ Department of Medicine, Lagos State University Teaching Hospital, Ikeja, Nigeria; ^5^ Department of Pharmaceutics and Pharmacy Practice, School of Pharmacy, University of Nairobi, Nairobi, Kenya; ^6^ Pharmaceutical Society of Kenya, Nairobi, Kenya; ^7^ Department of Pharmacology and Pharmacognosy, School of Pharmacy, University of Nairobi, Nairobi, Kenya; ^8^ Pharmacy Department, College of Medicine, Blantyre, Malawi; ^9^ Department of Pharmacy Practice and Policy, Faculty of Health Sciences, University of Namibia, Windhoek, Namibia; ^10^ Department of Pharmacology and Therapeutics, Faculty of Pharmaceutical Sciences, Bayero University, Kano, Nigeria; ^11^ Unit of Pharmacology, College of Health Sciences, Yusuf Maitama Sule University, Kano, Nigeria; ^12^ Faculty of Pharmacy, University of Lahore, Lahore, Pakistan; ^13^ Department of Pharmacy, University of Zambia, Lusaka, Zambia; ^14^ Pharmaceutical Administration and PharmacoEconomics, Hanoi University of Pharmacy, Hanoi, Vietnam; ^15^ Unit of Pharmacology, Faculty of Medicine and Defence Health, Universiti Pertahanan Nasional Malaysia (National Defence University of Malaysia), Kuala Lumpur, Malaysia; ^16^ Department of Microbiology, Jahangirnagar University, Dhaka, Bangladesh; ^17^ Department of Periodontology and Implantology, Karnavati University, Gandhinagar, India; ^18^ Healthcare Improvement Scotland, Glasgow, United Kingdom; ^19^ Uganda Alliance of Patients’ Organizations (UAPO), Kampala, Uganda; ^20^ Independent Consumer Advocate, Brunswick, VIC, Australia; ^21^ Institute of Orthopaedic Surgery “Banjica”, University of Belgrade, Belgrade, Serbia; ^22^ Strathclyde Institute of Pharmacy and Biomedical Sciences, University of Strathclyde, Glasgow, United Kingdom; ^23^ Department of Pharmacology, College of Pharmacy, Hawler Medical University, Erbil, Iraq; ^24^ Faculty of Health and Life Sciences, Brownlow Hill, Liverpool, United Kingdom; ^25^ QC Medica, York, United Kingdom; ^26^ School of Pharmacy, Sefako Makgatho Health Sciences University, Pretoria, South Africa; ^27^ School of Pharmaceutical Sciences, Universiti Sains Malaysia, Penang, Malaysia; ^28^ Division of Clinical Pharmacology, Karolinska Institute, Karolinska University Hospital Huddinge, Stockholm, Sweden

**Keywords:** Africa, Asia, community pharmacists, COVID-19, medicines, protective equipment, price rises, shortages

## Abstract

**Background:** Countries across Africa and Asia have introduced a variety of measures to prevent and treat COVID-19 with medicines and personal protective equipment (PPE). However, there has been considerable controversy surrounding some treatments including hydroxychloroquine where the initial hype and misinformation led to shortages, price rises and suicides. Price rises and shortages were also seen for PPE. Such activities can have catastrophic consequences especially in countries with high co-payment levels. Consequently, there is a need to investigate this further.

**Objective:** Assess changes in utilisation, prices, and shortages of pertinent medicines and PPE among African and Asian countries since the start of pandemic.

**Our approach:** Data gathering among community pharmacists to assess changes in patterns from the beginning of March until principally the end of May 2020. In addition, suggestions on ways to reduce misinformation.

**Results:** One hundred and thirty one pharmacists took part building on the earlier studies across Asia. There were increases in the utilisation of principally antimalarials (hydroxychloroquine) and antibiotics (azithromycin) especially in Nigeria and Ghana. There were limited changes in Namibia and Vietnam reflecting current initiatives to reduce inappropriate prescribing and dispensing of antimicrobials. Encouragingly, there was increased use of vitamins/immune boosters and PPE across the countries where documented. In addition, generally limited change in the utilisation of herbal medicines. However, shortages have resulted in appreciable price increases in some countries although moderated in others through government initiatives. Suggestions in Namibia going forward included better planning and educating patients.

**Conclusion:** Encouraging to see increases in the utilisation of vitamins/immune boosters and PPE. However, concerns with increased utilisation of antimicrobials needs addressing alongside misinformation, unintended consequences from the pandemic and any appreciable price rises. Community pharmacists and patient organisations can play key roles in providing evidence-based advice, helping moderate prices through improved stock management, and helping address unintended consequences of the pandemic.

## Introduction

We have recently published regarding ongoing activities across Africa to prevent and treat COVID-19 (Ogunleye et al., 2020). Activities to reduce the spread of COVID-19 were centered around measures to close borders, quarantine returning travellers and their contacts, restrict movement as well as track and trace citizens (Ogunleye et al., 2020). However, the various measures and initiatives were introduced at different times and with different intensities leading to differences in prevalence and mortality rates seen to date. We also see similar activities introduced among Asian countries including Bangladesh, Pakistan, and Vietnam (Table 1) with again variable impact on prevalence and mortality rates (Godman et al., 2020a; Haque et al., 2020a). Vietnam aggressively introduced a range of measures to prevent the spread of COVID-19 under the banner “Fighting the epidemic is like fighting again’t the enemy,” which together with other factors resulted in only 383 reported cases by 19 July and no deaths (Godman et al., 2020a; Jones, 2020; Pham et al., 2020; WHO, 2020a). This increased to 1,069 cases by 27 September and only 35 reported deaths (Godman et al., 2020a; WHO, 2020b). In Namibia in sub-Saharan Africa there were 1,247 reported cases and only 3 deaths up to July 19, 2020 following multiple measures (Table 1), although this is changing (Ogunleye et al., 2020; WHO, 2020b). In addition, in Ghana with its multiple activities there were only 148 recorded deaths from 27,667 confirmed cases by July 19, 2020 increasing to 46,222 confirmed cases by September 27, 2020 and 299 deaths, with Ghana seen as a model country for managing COVID-19 in sub-Saharan Africa (Ogunleye et al., 2020; WHO, 2020a; WHO, 2020b; Afriyie et al., 2020). However, it is recognised that there is severe under-reporting of prevalence rates and deaths in a number of countries due to low rates of testing and the robustness of reporting systems certainly initially (Godman et al., 2020a; Haque et al., 2020a; Ogunleye et al., 2020).

**TABLE 1:** Confirmed prevalence and mortality rates for COVID-19 among selected African and Asian countries (September 27, 2020).

Country	Population size	Prevalence rates reported	Current deaths	CFR rate (%)
Bangladesh	164,689,383	357,873	5,129	1.43
Ghana	31,072,940	46,222	299	0.65
Kenya	53,771,296	37,871	689	1.82
Malawi	19,129,952	5,766	179	3.10
Namibia	2,540,905	10,918	120	1.10
Nigeria	206,139,589	58,198	1,106	1.90
Pakistan	220,892,340	310,275	6,457	2.08
Vietnam	97,338,579	1,069	35	3.27
Zambia	18,383,955	14,612	332	2.27

NB, Population figures taken from Worldometer data for 2020 (Worldometer, 2020a; Worldometer, 2020b; Worldometer, 2020c; Worldometer, 2020d) and epidemiology data from WHO (WHO, 2020b). CFR, case fatality rate.

Table 1 documents current prevalence and mortality rates for a range of African countries where there can be high co-payments for medicines, as well as high rates of self-medication with antimicrobials despite regulations banning this, with potentially catastrophic consequences for some families in a number of countries when family members become ill (Kalungia et al., 2016; McHenga et al., 2017; Afari-Asiedu et al., 2018; Aregbeshola and Khan, 2018; Salari et al., 2019; Kaonga et al., 2019; Godman et al., 2020b). We also see a similar situation in Bangladesh and Pakistan with high co-payments levels and potentially catastrophic consequences for families when members become ill (Haque, 2017; Datta et al., 2019; Hsu et al., 2018; Pakistan Today, 2020). Increased social insurance coverage has reduced out-of-pocket payments in Vietnam; however, these still exist with potentially distressing consequences for a minority of families when members become ill. In addition, a high percentage of patients in Vietnam still visit pharmacists first for their illnesses (Pekerti et al., 2017; Hoa et al., 2019; Lee et al., 2019). The situation is different in Namibia with universal healthcare, provision of well accepted guidelines, monitoring of physician prescribing against current guidance as well as regulations banning the self-purchasing of antimicrobials (Nashilongo et al., 2017; Kamati et al., 2019; Niaz et al., 2019; Niaz et al., 2020).

Supplementary Table A1 in the Appendix summarises some of the activities that the chosen Asian countries have instigated to help reduce the spread of COVID-19. Activities among the selected African and Asian countries to reduce the spread of COVID-19 can be found in Godman et al. (2020a) and Ogunleye et al. (2020).

Whilst no medicine is currently fully recommended for treating COVID-19, there has been appreciable controversy surrounding the use of chloroquine and hydroxychloroquine with or without antibiotics including azithromycin (Ogunleye et al., 2020). There was considerable hype surrounding early reports regarding the effectiveness of hydroxychloroquine in reducing the impact of COVID-19 resulting in shortages, price rises, and deaths (Abena et al., 2020; Busari and Adebayo, 2020; Nga et al., 2020; Politi, 2020). However, there were concerns regarding the efficacy data with a lack of control arms in the early studies, endorsed by later studies showing no proven benefit from using hydroxychloroquine along with potential harm (Borba et al., 2020; Ferner and Aronson, 2020; ISAC/Elsevier, 2020; International Society of Antimicrobial Chemotherapy, 2020; Horby et al., 2020; Geleris et al., 2020; Littlejohn, 2020; WHO Solidarity Trial Consortium, 2020). Following these studies, the WHO halted the hydroxychloroquine arm in its ongoing Solidarity Trial with the National Institute of Health (NIH) in the United States also halting hydroxychloroquine arms in its studies (NIH, 2020; WHO, 2020c). Alongside this, in Africa the South African Pharmacy Council warned against the misuse of hydroxychloroquine for the treatment of COVID-19, building on concerns from the South African regulatory agency regarding the lack of evidence (SAHPRA, 2020; Masango, 2020). There have been similar concerns with lopinavir-ritonavir, with most studies failing to show clinical benefit including the United Kingdom (United Kingdom) Recovery study (RECOVERY Collaborative Group, 2020; Ford et al., 2020). As a result, the WHO has now also dropped lopinavir-ritonavir from the Solidarity trial (WHO Solidarity Trial Consortium, 2020; WHO, 2020c). There are now similar concerns with remdesivir (WHO Solidarity Trial Consortium, 2020; Dyer, 2020; McCreary and Angus, 2020; Wang et al., 2020). Early premature recommendations are a concern not only in rasing false hope among patients and physicians but also potentially exposing patients to severe adverse drug reactions as well as diverting valuable resources away from funding other priority disease areas. In addition, the over use of antimicrobials will enhance resistance rates, which is already a concern across Africa and Asia (Godman et al., 2020b). This includes hydroxychloroquine with artemetherlumefantrine or artesunate-amodiaquine now typically recommended for the treatment of uncomplicated malaria across Africa with hydroxychloroquine reserved due to resistance and other concerns (WHO, 2018; Conrad and Rosenthal, 2019).

Considerable price rises of antimalarials have already been seen in Bangladesh and Nigeria as a result of the hype surrounding hydroxychloroquine, which is a concern (Busari and Adebayo, 2020; Haque et al., 2020a). Considerable price rises have also been seen for personal protective equipement (PPE) in Bangladesh and Sudan, which again causes concern if increased spending on these items following price hikes reduces available resouces for food and medicines for other high priority disease areas (Weston, 2020).

There are also concerns with the inappropriate use of medicinal plants to prevent and treat COVID-19 as this could cause more harm than good (Ekor, 2014; Liwa et al., 2014; Nkeck et al., 2020; Nordling, 2020; Yang, 2020). We are aware some herbal medicines are showing promise in treating patients with COVID-19 based on *in vitro* and small-scale clinical studies (Ang et al., 2020; Luo E. et al., 2020; Luo L. et al., 2020; Vellingiri et al., 2020; Yang, 2020; Zhang et al., 2020). However, their use outside of formal trials is a concern until more data becomes available especially if taking these medicines causes delay in patients seeking more appropriate care (Yang, 2020), mirroring the controversies seen with hydroxychloroquine.

There are also unintended consequences from lockdown and other measures associated with the pandemic (Godman, 2020). These include concerns with the negative impact on immunisation and prevention programmes for infectious diseases as well as reduced control of non-communicable diseases (NCDs) such as cancer, coronary vascular diseases (CVD), hypertension and type 2 diabetes (T2DM) if patients fail to collect and/or take their medicines (Basu et al, 2020; Banerjee, 2020; Chandir et al., 2020; Kluge et al., 2020; UNICEF South Africa, 2020; WHO, 2020d). Recently Abbas *et al.* (2020) calculated that as a result of reduced immunisation programmes from lockdown and other activities, coupled with a lack of catch-up activities as lockdown measures ease, that for every one excess COVID-19 death acquired during routine vaccination clinic visits, 84 deaths in children could be avoided (Abbas et al., 2020). Equally, any appreciable reduction in the distribution of protective bed nets (75%), coupled with a lack of medicines to treat malaria due to lockdown and other activities, combined with lack of media campaigns, could result in up to 18 million additional cases and up to 30,000 additional deaths in sub-Saharan Africa alone compared to 2018 (Krubiner et al., 2020; Cash and Patel, 2020; WHO, 2020e).

Community pharmacists can play a key role helping with immunisation programmes as well as enhancing the appropriate use of antimicrobials. This is in addition to educating patients regarding effective strategies to prevent the spread of COVID-19 as well as help address misinformation especially surronding hydroxychloroquine and herbal medicines through posters, leaflets and other media. Alongside this, working with physicians and other groups to increase access to medicines for patients espeically those with chronic NCDs through mobile telephones and other technologies (Markovic-Pekovic et al., 2017; Ahmad et al., 2020; Cadogan and Hughes, 2020; Dzingirai et al., 2020; Erku et al., 2020; FIP, 2017; FIP, 2020; Godman et al., 2020b; Kretchy et al., 2020). However, community pharmacists need to be mindful of their own potential for catching the virus and taking appropriate precautions including when re-stocking from suppliers (Dzingirai et al., 2020; Ogunleye et al., 2020). Pharmacy groups can also help address shortages through proactive measures including better stock management, which could be critical for medicines to treat malaria and other priority disease areas across Africa. This is because medicine shortages are an increasing concern among African countries where typically up to 94% of medicines are imported (N Gage Consuting, 2017; Bavier, 2020; Dugmore, 2020). This is leading to calls to increase local production, building on initiatives to address the lack of ventilators and testing equipment at the start of the pandemic in Africa, with such calls likely to continue post pandemic (EAC, 2020; GhanaWeb, 2020a; Ogunleye et al., 2020).

Consequently, we believe there is a need to rapidly determine changes in utilisation and prices as well as shortages of recommended medicines and PPE for the prevention and treatment of COVID-19 in the early stages of the pandemic especially among African countries to help guide future strategies. This includes changes in utilisation and prices of hydroxychloroquine, antibiotics especially azithromycin, and herbal medicines arising from any early endorsement. We are aware that patient groups are important to convey correct information about effective preventative measures as well as address misinformation including misconceptions about vaccines and the consequences (Anna, 2020; Ogunleye et al., 2020). We are also aware that groups such as the International Alliance of Patients’ Organizations (IAPO) have developed resource hubs to provide reliable and updated information to mitigate against misinformation and promote effective preventative activities (International Alliance of Patients’ Organizations, 2020). In addition, patient groups are vital to help reduce any stigma associated with COVID-19 (AFP-JIJI, 2020; Kamal, 2020; IFRC, UNICEF and WHO, 2020; GhanaWeb, 2020b; He et al., 2020; Ogunleye et al., 2020). However, this is outside of this current project.

## Materials and Methods

We adopted a similar comprehensive strategy applied in Bangladesh (Haque et al., 2020a), and subsequently used among other Asian countries (Godman et al., 2020a; Haque et al., 2020b), building on previous knowledge of activities across Africa just before and after the first cases of COVID-19 were identified in Africa (Ogunleye et al., 2020). This included a questionnaire survey among community pharmacies in Africa including Ghana, Kenya, Namibia, Malawi, Nigeria, and Zambia as well as among three comparative Asian countries including Bangladesh, Pakistan and Vietnam. **Box 1** summarises the open ended questions building on published studies (Haque et al., 2020a; Haque et al., 2020b). Impressions were requested if this was the only information available due to issues of confidentiality and culture as no payment was made to pharmacists for the information provided (Godman et al., 2020a; Haque et al., 2020a). The objective was to assess the current situation regarding usage patterns, prices, and availability of selected medicines and PPE used in prevention and management of COVID-19 soon after the start of the pandemic. The baseline was early 2020, i.e., just before active preventative measures in a number of the countries. More specific data on actual changes in utilisation and prices was asked if this was available; however, this did not include asking the pharmacists to break down changes in utilisation patterns and prices per month as this was deemed too problematic for this initial study. In addition, it was envisaged there would be limited impact of seasonality in view of year-round influenza activity in some African countries and no real pattern in others (WHO, 2012; Dawa et al., 2020; WHO Regional Office for Africa, 2020) although research is ongoing in this area to address current knowledge gaps (Sambala et al., 2018). In addition, generally limited use of hydroxychloroquine in immunological diseases such as rheumatoid arthritis with concerns over its effectiveness compared with other disease modifying therapies (Rempenault et al., 2020). A more detailed description of the questionnaire can be found in Haque et al. (2020a).

BOX 1 **Open ended questions to community pharmacists across Africa regarding pertinent medicines and equipment to prevent and treat COVID-19.**

- Country?
- What changes in purchasing/utilisation patterns have you noticed in your pharmacy from the beginning of March, i.e. with the first cases being recorded and just before lockdown and other measures typically introduced, until the end of May 2020 for antimalarials (hydroxychloroquine), antibiotics (e.g. azithromycin), multivitamins including vitamin C, analgesics and herbal medicines (where pertinent)? The baseline is early 2020, i.e. pre the pandemic. Please base this information on invoices where possible or other information sources; otherwise, impressions
- What changes in the prices have you noticed for these medicines from the beginning of March until end May 2020 – again based on invoices or other information sources where possible; otherwise impressions. The baseline is again early 2020
- Have there been any shortages for these pertinent medicines from the beginning of March until end May 2020 ? If so, what has been the extent if known?
- Similarly, for PPE including face masks and other equipment such as hand sanitisers from the beginning of March until the end May 2020 (baseline early 2020) - based again on invoices or other information sources/impressions where possible
- What suggestions can you give the authorities to address misinformation regarding the current pandemic (if pertinent) as well as any inappropriate self-medication with antimicrobials for future pandemics given current concerns?

We did not factor inflation into the impressions as the study period only covered a short time, and per annum inflation in the chosen African countries typically ranged between 6 and 16% per annum (Focus Economics, 2020; Trading Economics, 2020a; Trading Economics, 2020b; Trading Economics, 2020c; Trading Economics, 2020d; Trading Economics, 2020e).

The Asian countries chosen for comparative purposes had instigated similar activities to prevent the spread of COVID-19 as those seen in Africa and where there can be high patient co-payments for medicines (Godman et al., 2020a).

Convenience sampling was again used to select pharmacists through emails, telephone contact, personal contacts and other mechanisms similar to the studies across Asia (Godman et al., 2020a; Haque et al., 2020a). As before, there was no sample size calculation as there was no previous data to base calculations upon at the start of the study and this was a pilot study with a minimum of six pharmacies contacted in all countries apart from Namibia to determine the need for additional follow-up studies.

The findings were subsequently consolidated into categories in a tabular format to aid comparisons between regions and countries, with more specific data available in country publications (Haque et al., 2020a; Haque et al., 2020b). We believed that there would be price rises and shortages in other countries apart from Bangladesh. However, the nature and extent would depend on ongoing programmes within the country.

We also explored the situation regarding the preparedness of community pharmacists in Namibia to the pandemic to help enhance future guidance to Governments and other key stakeholder groups. This included questions on i) Key measures/interventions the pharmacy has put in place during the pandemic to curb the spread of COVID-19 in the community (maximum of three from a pre-arranged list of seven known activities); ii) Suggestions on the role (current and new) of pharmacists/pharmaceutical technicians/pharmacist assistants during current and future pandemics (up to three from a pre-arranged list of five potential activities); iii) The main challenges experienced by pharmacy personnel during the pandemic (maximum of three from a pre-arranged list of seven known activities); iv) Changes in utilization, prices and shortages of pertinent medicines and PPE used in the prevention and management of COVID-19 from the beginning of March to end June 2020 (Supplementary Appendix A1). The study was extended to the end of June to provide greater insight.

Potential future guidance for governments, pharmacists and patients will build on the experiences of the pharmacists and others involved in the study in Namibia and across all the studied countries, the co-authors and previous suggestions documented in Ogunleye et al. (2020).

Ethical approval for this study was not required according to our national legislation and institutional guidelines. However, as before in Bangladesh, all pharmacists freely provided the requested information having been given the opportunity to refuse to participate. This is in line with previous studies undertaken by the co-authors in this and related areas including analysis of policies to enhance the use of biosimilars and the rationale use of medicines, pricing policies as well as issues surrounding shortages and generics, which typically involved direct contact with health authority personnel and other key stakeholders (Godman et al., 2014; Godman et al., 2015; Moorkens et al., 2017; Godman et al., 2019; Gad et al., 2020; Godman et al., 2020a; Godman et al., 2020b; Godman et al., 2020c; Godman et al., 2020d; Haque et al., 2020a; Miljković et al., 2020a).

## Results

Table 2 documents the number of community pharmacists involved in Ghana, Kenya, Namibia, Malawi, Nigeria, and Zambia as well as Pakistan and Vietnam building on the published paper of Haque et al. (2020a) and Godman et al. (2020b). As mentioned, typically pilot studies were conducted in all countries apart from Namibia to provide direction for future studies if needed building on this rapid pilot study.

**TABLE 2:** Details of pharmacists and drug stores owners across the countries.

Country	Number of pharmacies
Ghana	6
Kenya	6
Malawi	11
Namibia	55
Nigeria	30
Pakistan	9
Vietnam	6
Zambia	8
Total	131

### Changes in Utilisation Patterns

Table 3 depict changes in the utilisation patterns for the various medicines, vitamins, and PPE (where documented) from the beginning of March until the end of May 2020 among the studied countries.

**TABLE 3:** Utilisation changes (percentages) for medicines and PPE between beginning March 2020 and end May 2020 among pharmacies across the countries.

Change	Ghana					Kenya					Malawi				
	AM	AB	AG	Vit C	HM	AM	AB	AG	Vit C	HM	AM	AB	AG	Vit C	HM
Decrease/No or low demand (%)	0.0	0.0	0.0	0.0	0.0	16.7	0.0	0.0	0.0	0.0	0.0	0.0	9.1	0.0	0.0
No change/not sold/not specified (%)	16.7	0.0	83.3	0.0	0.0	16.7	33.3	33.3	0.0	100.0	45.5	63.6	90.9	27.3	54.5
Slight increase to increase (not specified) (%)	0.0	83.3	0.0	0.0	0.0	66.7	66.7	50.0	100.0	0.0	45.5	18.2	0.0	27.3	18.2
High increase/high demand to under 1.5 fold increase (%)	83.3	16.7	16.7	100.0	100.0	0.0	0.0	16.7	0.0	0.0	0.0	9.1	0.0	18.2	18.2
1.5 fold–3 fold increase (%)	0.0	0.0	0.0	0.0	0.0	0.0	0.0	0.0	0.0	0.0	9.1	9.1	0.0	27.3	9.1
3 to 5 fold increase (%)	0.0	0.0	0.0	0.0	0.0	0.0	0.0	0.0	0.0	0.0	0.0	0.0	0.0	0.0	0.0
Above 5 fold increase (%)	0.0	0.0	0.0	0.0	0.0	0.0	0.0	0.0	0.0	0.0	0.0	0.0	0.0	0.0	0.0
Total (Percentage)	100	100	100	100	100	100	100	100	100	100	100	100	100	100	100
% Increase	83.3	100.0	16.7	100.0	100.0	66.7	66.7	66.7	100.0	0.0	54.5	36.4	0.0	72.7	45.5
% No change/decrease	16.7	0.0	83.3	0.0	0.0	33.3	33.3	33.3	0.0	100.0	45.5	63.6	100.0	27.3	54.5

Change	Nageria					Namibia						Zambia				
	AM	AB	AG	Vit C	PPE	AM	AB	AG	Vit C	HM	PPE	AM	AB	AG	Vit C	HM
Decrease/No or low demand (%)	0.0	0.0	0.0	0.0	0.0	0.0	0.0	0.0	0.0	0.0	0.0	25.0	25.0	25.0	25.0	33.3
No change/not sold/not specified (%)	0.0	13.3	93.3	0.0	0.0	81.8	72.7	58.2	23.6	80.0	0.0	0.0	25.0	0.0	12.5	11.1
Slight increase to increase (not specified) (%)	0.0	0.0	3.3	0.0	0.0	1.8	10.9	14.5	3.6	5.5	1.8	37.5	25.0	25.0	37.5	33.3
High increase/high demand to under 1.5 fold increase (%)	0.0	0.0	0.0	0.0	0.0	10.9	5.5	21.8	40.0	12.7	10.9	37.5	25.0	50.0	25.0	22.2
1.5 fold–3 fold increase (%)	0.0	40.0	0.0	3.3	0.0	5.5	10.9	5.5	32.7	1.8	87.3	0.0	0.0	0.0	0.0	0.0
3 to 5 fold increase (%)	30.0	43.3	3.3	26.7	13.3	0.0	0.0	0.0	0.0	0.0	0.0	0.0	0.0	0.0	0.0	0.0
Above 5 fold increase (%)	70.0	3.3	0.0	70.0	86.7	0.0	0.0	0.0	0.0	0.0	0.0	0.0	0.0	0.0	0.0	0.0
Total (Percentage)	100.0	100	100	100	100	100	100	100	100	100	100	100	100	100	100	100
% Increase	100.0	86.7	6.7	100.0	100.0	18.2	27.3	41.8	76.4	20.0	100.0	75.0	50.0	75.0	62.5	55.6
% No change/decrease	0.0	13.3	93.3	0	0.0	81.8	72.7	58.2	23.6	80.0	0.0	25.0	50.0	25.0	37.5	44.4

Change	Bangladesh					Pakistan					Vietnam					
	AM	AB	AG	Vit C	PPE	AM	AB	AG	Vit C	HM	AM	AB	AG	Vit C	HM	PPE
Decrease/No or low demand (%)	5.3	2.9	0.0	0.0	1.2	0.0	0.0	0.0	11.1	0.0	0.0	100.0	66.7	33.3	100.0	0.0
No change (%)	45.9	26.5	2.4	9.4	3.5	11.1	0.0	55.6	0.0	66.7	100.0	0.0	0.0	0.0	0.0	0.0
Sight increase to increase (not specified) (%)	42.9	61.2	83.5	82.4	81.8	55.6	77.8	33.3	33.3	33.3	0.0	0.0	33.3	66.7	0.0	0.0
High increase to under 1.5 fold increase (%)	0.6	5.3	8.8	2.9	5.9	22.2	11.1	11.1	44.4	0.0	0.0	0.0	0.0	0.0	0.0	100.0
1.5 fold–3 fold increase (%)	5.3	4.1	5.3	5.3	7.6	11.1	11.1	0.0	11.1	0.0	0.0	0.0	0.0	0.0	0.0	0.0
3 to 5 fold increase (%)	0.0	0.0	0.0	0.0	0.0	0.0	0.0	0.0	0.0	0.0	0.0	0.0	0.0	0.0	0.0	0.0
Above 5 fold increase (%)	0.0	0.0	0.0	0.0	0.0	0.0	0.0	0.0	0.0	0.0	0.0	0.0	0.0	0.0	0.0	0.0
Total percentage	100.0	100.0	100.0	100.0	100.0	100.0	100.0	100.0	100.0	100.0	100.0	100.0	100.0	100.0	100.0	100.0

NB, AM, antimalarials (principally hydroxychloroquine); AB, Antibiotics (principally azithromycin); AG, analgesics; Vit C, Vitamin C and other Vitamins and immune boosters; HM, herbal medicines, PPE, Personal Protective Equipment including face masks and hand sanitisers. No change also includes situations where medicines were not dispensed without a prescription or not dispensed in community pharmacies (antimalarials and antibiotics). NB, Namibia up to end June 2020.

There was an increase in the utilisation of antimalarials (principally hydroxychloroquine) among all the African countries, greatest in Ghana, Nigeria and Zambia (Table 3). This is similar to Pakistan although increases appear less than seen in Ghana and Nigeria, but appreciably different to Vietnam. There were also increases in the utilisation of antibiotics (principally azithromycin) among all the African countries, greatest in Ghana, Kenya and Nigeria. There was also increased utilisation of antibiotics in Pakistan vs. Bangladesh and Vietnam. There was generally more limited increase in the utilisation of analgesics across countries, with a decrease seen in Vietnam. Encouragingly, there was an increase in the utilisation of vitamins and immune boosters across countries, and similarly for PPE (Table 3). There were variable increases in the utilisation of herbal medicines among the studied countries, greatest in Ghana vs. no or limited change in Kenya and Namibia.

### Price Changes

Table 4 depicts price changes for pertinent medicines and PPE during the study period. Again, there were differences between countries reflecting differences in utilisation rates (Table 3) and shortages (Table 5).

**TABLE 4:** Price changes for medicines and PPE between beginning March 2020 and end May 2020 among pharmacies across the countries.

Change	Ghana					Kenya				Malawi				
	AM	AB	AG	Vit C	HM	AM	AG	Vit C	HM	AM	AB	AG	Vit C	HM
Data not available/decrease (%)	0.0	0.0	0.0	0.0	0.0	0.0	0.0	0.0	0.0	0.0	0.0	0.0	0.0	0.0
No change/not sold (%)	50.0	66.7	66.7	16.7	50.0	50.0	50.0	0.0	100.0	72.7	81.8	90.9	45.5	100.0
Increase (no specified)/slight increas (%)	33.3	33.3	33.3	66.7	16.7	0.0	0.0	0.0	0.0	0.0	9.1	0.0	45.5	0.0
High increase up to 2 fold increase (%)	0.0	0.0	0.0	16.7	33.3	50.0	50.0	100.0	0.0	18.2	9.1	9.1	9.1	0.0
2–4 fold increase (%)	16.7	0.0	0.0	0.0	0.0	0.0	0.0	0.0	0.0	9.1	0.0	0.0	0.0	0.0
Above 4 fold increase (%)	0.0	0.0	0.0	0.0	0.0	0.0	0.0	0.0	0.0	0.0	0.0	0.0	0.0	0.0
Total (percentage)	100	100	100	100	100	100	100	100	100	100	100	100	100	100
% Increase	50.0	33.3	33.3	83.3	50.0	50.0	50.0	100.0	0.0	27.3	18.2	9.1	54.5	0.0
% No change/decrease	50.0	66.7	66.7	16.7	50.0	50.0	50.0	0.0	100.0	72.7	81.8	90.9	45.5	100.0

Change	Nageria						Namibia					Zambia				
	AM	AB	AG	Vit C	HM	PPE	AM	AB	AG	Vit C	PPE	AM	AB	AG	Vit C	HM
Data not available/decrease (%)	0.0	0.0	0.0	0.0	0.0	0.0	0.0	0.0	0.0	0.0	0.0	0.0	0.0	0.0	0.0	0.0
No change/not sold/not record (%)	96.4	94.5	92.7	69.1	96.4	52.7	3.3	6.7	86.7	33.3	0.0	0.0	12.5	12.5	12.5	75.0
Increase (no specified)/slight increase (%)	1.8	1.8	5.5	10.9	3.6	0.0	0.0	0.0	13.3	0.0	0.0	62.5	62.5	62.5	37.5	25.0
High increase upto 2 fold increase (%)	1.8	3.6	1.8	20.0	0.0	41.8	0.0	93.3	0.0	66.7	0.0	37.5	25.0	25.0	50.0	0.0
2–4 fold increase (%)	0.0	0.0	0.0	0.0	0.0	5.5	50.0	0.0	0.0	0.0	0.0	0.0	0.0	0.0	0.0	0.0
Above 4 fold increase (%)	0.0	0.0	0.0	0.0	0.0	0.0	46.7	0.0	0.0	0.0	100.0	0.0	0.0	0.0	0.0	0.0
Total (percentage)	100	100	100	100	100	100	100	100	100	100	100	100	100	100	100	100
% Increase	3.6	5.5	7.3	30.9	3.6	47.3	96.7	93.3	13.3	66.7	100.0	100.0	87.5	87.5	87.5	25.0
% No change/decrease	96.4	94.5	92.7	69.1	96.4	52.7	3.3	6.7	86.7	33.3	0.0	0.0	12.5	12.5	12.5	75.0

Change	Bangladesh					Pakistan					Vietnam					
	AM	AB	AG	Vit C	PPE	AM	AB	AG	Vit C	HM	AM	AB	AG	Vit C	HM	PPE
Data not available/decrease (%)	2.4	0.0	0.0	0.0	0.6	0.0	0.0	0.0	0.0	0.0	100.0	0.0	0.0	0.0	0.0	0.0
No change (%)	47.6	65.3	54.7	51.8	4.1	88.9	77.8	88.9	77.8	88.9	0.0	83.3	66.7	83.3	100.0	0.0
Slight increase to increase (unspecified) (%)	32.9	25.9	34.1	42.9	64.7	11.1	22.2	11.1	22.2	11.1	0.0	16.7	33.3	16.7	0.0	100
HIgh increase up to 2 fold increase (%)	11.8	8.2	10.6	5.3	20.0	0.0	0.0	0.0	0.0	0.0	0.0	0.0	0.0	0.0	0.0	0.0
2–4 fold increase (%)	5.3	0.6	0.6	0.0	8.8	0.0	0.0	0.0	0.0	0.0	0.0	0.0	0.0	0.0	0.0	0.0
Above 4 fold increase (%)	0	0	0.6	0	1.8	0.0	0.0	0.0	0.0	0.0	0.0	0.0	0.0	0.0	0.0	0.0
Total %	100.0	100.0	0	100.0	100.0	100.0	100.0	100.0	100.0	100.0	100.0	100.0	100.0	100.0	100.0	100.0

NB, AM, antimalarials (principally hydroxychloroquine); AB, Antibiotics (principally azithromycin); AG, analgesics; Vit C, Vitamin C, other Vitamins and other immune boosters; HM, Herbal medicines; PPE, Personal Protective Equipment such as face masks and hand sanitisers.

NB, Namibia up to end June 2020.

**TABLE 5:** Shortages for medicines and PPE between beginning March 2020 and end May 2020 among pharmacies across the countries.

Change	Ghana					Kenya					Malawi				
	AM	AB	AG	Vit C	HM	AM	AB	AG	Vit C	HM	AM	AB	AG	Vit C	HM
Available/no shortage recorded/not dispensed (%)	50.0	83.3	100.0	0.0	33.3	100.0	100.0	83.3	100.0	100.0	54.5	81.8	81.8	50.0	90.9
Shortage (unspecified) including substained (%)	16.7	0.0	0.0	66.7	0.0	0.0	0.0	16.7	0.0	0.0	27.3	18.2	0.0	20.0	0.0
Shortages (some of the time) - percentages	33.3	16.7	0.0	33.3	66.7	0.0	0.0	0.0	0.0	0.0	18.2	0.0	18.2	30.0	9.1
Total (percentages)	100	100	100	100	100	100	100	100	100	100	100	100	100	100	100
% Shortages (% of total)	50.0	16.7	0.0	100.0	66.7	0.0	0.0	16.7	0.0	0.0	45.5	18.2	18.2	50.0	9.1

Change	Nageria						Namibia					Zambia				
	AM	AB	AG	Vit C	HM	PPE	AM	AB	AG	Vit C	PPE	AM	AB	AG	Vit C	HM
Available/no shortages recorded/not dispensed (%)	69.1	69.1	89.1	56.4	90.9	23.6	26.7	90.0	96.7	6.7	20.0	25.0	62.5	62.5	37.5	50.0
Shortages (unspecified) including substaintial (%)	30.9	30.9	10.9	43.6	9.1	0.0	0.0	10.0	3.3	6.7	80.0	75.0	37.5	37.5	62.5	50.0
Shortages (some of the time) (%)	0.0	0.0	0.0	0.0	0.0	76.4	73.3	0.0	0.0	86.7	0.0	0.0	0.0	0.0	0.0	0.0
Total (percentage)	100	100	100	100	100	100	100	100	100	100	100	100	100	100	100	100
% Shortages (% of total)	30.9	30.9	10.9	43.6	9.1	76.4	73.3	10.0	3.3	93.3	80.0	75.0	37.5	37.5	62.5	50.0

Change	Bangladesh					Pakistan^a^					Vietnam					
	AM	AB	AG	Vit C	PPE	AM	AB	AG	Vit C	HM	AM	AB	AG	Vit C	PPE	HMs
Available/no shortages/not dispensed (%)	45.9	82.4	75.9	61.8	20.0	11.1	22.2	88.9	22.2	100.0	100.0	66.7	100.0	100.0	0.0	100.0
Shortages (unspecified/not available (%)	48.8	11.8	21.8	33.5	71.8	55.6	66.7	11.1	44.4	0.0	0.0	33.3	0.0	0.0	0.0	0.0
Shortages - some of the time (%)	5.3	5.9	2.4	4.7	8.2	33.3	11.1	0.0	33.3	0.0	0.0	0.0	0.0	0.0	100.0	0.0
Total (percentage)	100.0	100.0	100.0	100.0	100.0	100.0	100.0	100.0	100.0	100.0	100.0	100.0	100.0	100.0	100.0	100.0
% Shortages	54.1	17.6	24.1	38.2	80.0	88.9	77.8	11.1	77.8	0.0	0.0	33.3	0.0	0.0	100.0	0.0

NB, AM, antimalarials; AB, antibiotics; AG, analgesics; Vit C, Vitamin C, other Vitamins and other immune boosters; HM, Herbal medicines; PPE, Face masks, thermometers and hand sanitisers.

a Shortages includes decrease availability. N, Namibia up to end June 2020.

There were price rises for antimalarials (hydroxychloroquine) in an appreciable number of participating pharmacies in Nigeria and Zambia during the study period, with price rises in Ghana, Kenya and Malawi some of which were appreciable. Price rises were also seen in some of the pharmacies in Bangladesh, some of which were substantial; however, not in Pakistan nor Vietnam (Table 4). There was a similar picture for antibiotics (azithromycin). We also saw price increases for analgesics in Kenya and Zambia, some of which were appreciable. However, limited or no change in most of the pharmacies in the studied countries including the three Asian countries. There were also price increases for vitamin C/immune boosters across countries, some of which were substantial, e.g., Nigeria. There were price increases for PPE among all the studied countries (Table 4).

### Extent of Any Shortages

Perhaps not surprisingly, shortages were seen for a number of the surveyed medicines across Africa, especially antimalarials (hydroxychloroquine) and vitamin C/immune boosters, mirroring the situation among the participating Asian countries (Table 5). However to a lesser extent for antibiotics (principally azithromycin) and analgesics across the studied countries (Table 5). Where recorded, shortages of PPE were seen in all countries including Kenya and Malawi (not shown as not recorded in all pharmacies visited) influencing the extent of price rises seen in practice (Table 4).

### Experiences of Pharmacists in Namibia Regarding Their Preparedness and Activities to Reduce the Spread of COVID-19

Fifty five public and private pharmacists from 10 out of the 11 regions in Namibia took part among rural (18.2% of pharmacists), semi-urban (29.1%) and urban settings (52.7% of all pharmacists). Among these, 15 out of 55 (27.3%) pharmacists were prepared for the pandemic. All of these 15 pharmacies bulk ordered medicines, sanitizers/disinfectants and PPE in anticipation of increased demand with two out of the 15 (13.3%) pharmacies also making arrangements for enough staff to be available to cope with the anticipated increase in demand.

Pharmacists had put in place a number of measures to help curb the spread of COVID-19 with promoting prevention including hand sanitization and social distancing key suggested activities. These are described in Table 6 with all pharmacists (55 in all) putting in place at least one measure, 51 (92.7%) pharmacists instigating at least two measures and 23 pharmacists (41.8%) three measures – with only a maximum of three asked in the questionnaire.

**TABLE 6:** Activities instigated by pharmacists to prevent the spread of COVID-19.

Activity	Number
Promiting sanitization/disinfection/handwashing	41
Social distancing protocols	30
Promoting personal protective equipment	29
Temperature screening before entry	6
Name on a register	6
Multi-month dispensing of medicines	13
Creating awareness	4

NB, The total adds up to more than 55 as pharmacists can undertake multiple activities.

Table 7 contains suggestions from pharmacists, pharmaceutical technicians, and pharmacist assistants on their current and future potential roles to reduce the spread of any virus during a pandemic, with educating patients and advising on potential treatment plans key activities going forward. 53 pharmacists (96.4%) suggested one role, 28 (50.1%) two roles and one pharmacist three roles.

**TABLE 7:** Suggestions on the role of pharmacists/pharmaceutical technicians/pharmacist assistant during this pandemic.

Activity	Number
Educating and providing information on Covid-19 to the public	27
Advising on treatment plan as well as providing the medicines	24
Screening of patients that present to pharmacy and referring them for further testing if necessary	7
Supply PPE and Sanitizers	14
Stock control	10

NB, The total adds up to more than 55 as pharmacists can propose multiple suggestions.

Table 8 discusses the principal challenges experienced by pharmacy personnel in Namibia during the pandemic. 54 (98.2%) pharmacists documented one challenge, 26 (47.3%) two challenges and two pharmacists (3.6%) three challenges. Not surprisingly, inadequate stock of PPE and other preventative measures were key challenges building on observations documented in Table 6.

**TABLE 8:** The main challenges experienced by pharmacy personnel during the pandemic.

Activity	Number
Inadequate stock of PPE	41
Medicine stock-outs/low stock	30
Shortage of sanitizers/disinfectants	29
Inadequate space in pharmacy for staff to maintain social distancing	6
Increased workload due to initial panic buying and increased demand of items	6
Clinets/staff not adhering to regulations	13
Increments in medicine prices hence impact on finances	4

NB, The total adds up to more than 55 as pharmacists can suggest multiple challenges.

### Potential Ways Forward Among Key Stakeholder Groups

Potential ways forward for all key stakeholder groups are contained in Table 9, building on comments in Tables 7 and 8 as well as the feedback from other community pharmacists and the co-authors from across Africa and other countries.

**TABLE 9:** Potential activities among stakeholder groups to improve prevention and management of patients with COVID-19.

Stakeholder group	Suggested activities
Government	Encourage the early instigation of preventative measures with all groups to reduce the spread of any virus during current and future pandemics. This builds on the successes to date with limiting prevalence rates and mortality from COVID-19 in Botswana, Namibia and Vietnam vs. for instance Bangladesh and Pakistan [Table 1, Godman et al. (2020a), Haque et al. (2020a), Ogunleye et al. (2020)]
	All pertinent platforms should be used to rapidly broadcast effective methods to reduce transmission of the virus including social media and community pharmacists to rapidly disseminate information. Such tactics appear to be working as seen for instance in Pakistan where 77.0% of citizens in a recent survey believed COVID-19 could be controlled with practices such as wearing masks (85.8%) and handwashing (88.1%) (Hayat et al. 2020), with similar findings in Nepal (Singh et al. 2020) and Paraguay (Rios-González 2020). Such activities can also help reduce the stigma associated with COVID-19, which is a concern across countries (Bagcchi (2020), He et al. (2020), GhanaWeb (2020b), IFRC, UNICEF and WHO (2020))
	Governments need to be mindful of the ability of its citizens to social distance and have regular access to clean water in any deliberations - especially in the slums/overcrowded cities in LMICs Abdullah et al. (2020)), (Godman et al. (2020a), Haque et al. (2020a), Saaliq (2020)
	Governments also need to ensure healthcare facilities encourage the use of PPE among all staff members aware that social distancing can be difficult among certain facilities
	Encourage an evidence-based approach among all key decision makers to recommended measures to prevent and treat COVID-19 and subsequent pandemics, resisting the urge for early broadcasting of potential treatments without sound clinical data given the controversies surrounding hydroxychloroquine, lopinavir/ritonavir, and remdsivir and their impact, current controversies surrounding BCG vaccination as possible protection against COVID-19 (WHO Solidarity Trial Consortium (2020), O’Neill and Netea (2020), Schaaf et al. (2020)), concerns with trial design of a number of studies as well as the redaction of a number of recent papers relating to COVID-19 (International Society of Antimicrobial Chemotherapy (2020), ISAC/Elsevier (2020), Mehta et al. (2020), ECDC (2020), The Lancet editors (2020), Bae et al. (2020)). This can be helped by having a sound pro-active action plan to deal with the pandemic among all key stakeholder groups – building on the experiences of pandemics in countries such as Vietnam (Godman et al. (2020a))
	Instigate measures, including potential fines or imprisonment, to reduce the level of misinformation and its effects on diverting scarce resources away from funding as well as using medicines in other priority infectious and non-infectious diseases given the impact of the unintended consequences of COVID-19 (Kluge et al. (2020), Thornton (2020)). This builds on activities among a number of countries (Davis (2020), Dzirutwe (2020), Ogunleye et al. (2020)), and is in line with advice and recommendations from the Council for International Organisations of Medical Sciences (Council for International Organizations of Medical Sciences 2020).
	Vital to ensure as far as possible that ongoing and planned programmes to improve the management of patients with existing chronic NCDs across LMICs are continued so care is not compromised given concerns with the impact of lockdown and other measures on future morbidity and mortality of NCDs such as CVD and T2DM (Basu (2020), Kluge et al. (2020), Ogunleye et al. (2020))
	Similarly, for vaccination programmes given concerns with the potential impact on morbidity and mortality among children if programmes are halted/compromised (Abbas et al. (2020), Banerjee (2020), Chandir et al. (2020))
	Where pertinent, seek to enhance measures to reduce inappropriate self-purchasing of antimicrobials and other medicines of concern, building on successful measures in other countries including pharmacist and patient education and possible fines (Mukokinya et al. (2018), Jacobs et al. (2019), Alrasheedy et al. (2020), Godman et al. (2020b)) along with instigating antimicrobial stewardship programmes in ambulatory care
	Seek to enhance the local production of medicines, equipment, and PPE to address current and future shortages, as well as help keep prices rises to a minimum, mindful of concerns with the quality of locally produced medicines among some LMICs (Fadare et al. (2016), Khan et al. (2016)). In addition evaluate possible formal price controls building on examples in India and Pakistan (Lee et al. (2017), Haque et al. (2020b))
	Alongside this, introduce programmes if needed to reduce possible shortages of priority medicines and equipment building on experiences in other countries including recommending substitute medicines where suitable acknowledging the risks (Acosta et al. (2019), Chigome et al. (2019), Miljković et al. (2020a), Miljković et al. (2020), Modisakeng et al. (2020))
	When pertinent, adopt a phased approach to any easing of lockdown and other measures to help reduce possible spikes with increasing rates, with rapid re-introduction if needed Habersaat et al. (2020)
Pharmacists	Continue to encourage through education and other approaches a number of strategies to prevent the spread of COVID-19 building on Table 6, which includes preventative measures such as PPE and screening of patients, mindful of the need to wear PPE and social distance where possible (Cadogan and Hughes (2020), FIP (2020), Kretchy et al. (2020)). This may mean employing more staff during the early stages of a pandemic to cope with increased demand for medicines and advice (*Experiences of Pharmacists in Namibia Regarding Their Preparedness and Activities to Reduce the Spread of COVID-19*)
	Encourage patients to seek testing and medical advice where COVID-19 is suspected building on current activities in Vietnam with pharmacists asking patients buying medicines for acute respiratory infections such as coughs, fever, shortness of breath and colds to make a health declaration (Haque et al. (2020b), Vietnam Insider (2020))
	Try and ensure PPE and pertinent medicines are routinely available through pro-actively improving stock control as seen among some pharmacies in Namibia (*Experiences of Pharmacists in Namibia Regarding Their Preparedness and Activities to Reduce the Spread of COVID-19*). As part of this, help ensure where possible that any price rises are kept to a minimum especially in countries with high co-payment levels
	Discourage self-purchasing of antimicrobials to reduce resistance development acknowledging it can be difficult in practice to differentiate respiratory tract infections from COVID-19 among patients presenting with coughs and fever (Ongole et al. 2020)
	Work with patients with chronic NCDs to increase their supply of medicines (Table 6) and enhance adherence to these especially where it can be difficult for patients to attend clinics; alongside this work with patients where possible to help address issues of mental health (Basu (2020), Hayden and Parkin (2020))
	Potentially become involved in vaccination programmes given concerns with the impact on future morbidity and mortality if children fail to be vaccinated (Aruru et al. (2020), Ogunleye et al., (2020), WHO (2020))
Patients/Patient organisations	Patients and patient organisations should seek to promote via social media and other channels evidence-based approaches to the prevention and management of COVID-19 given the current extent of misinformation to date and the potential devastating consequences, ensuring that any messages are as clear as possible and in a positive language (Habersaat et al. 2020). Such approaches have helped in Pakistan, Nepal and Paraguay with good patient understanding of prevention activities (Hayat et al. (2020a), Singh et al. (2020), Rios-González (2020))
	As part of this, try to work with governments and other key stakeholder groups to minimise the impact of any misinformation (Habersaat et al. 2020) as well as any stigma associated with COVID 19 for healthcare professionals and patients
	Continue to work with key professional groups surrounding ways to reduce mental health problems arising from COVID-19 and its consequences
	Educate patients through various channels on ways to continuing managing chronic NCDs including CVD and T2DM including the importance of self-management and adherence to prescribed treatments given the challenges associated with lockdown activities on clinic attendance and collection of medicines, and potential devastating consequences (Kluge et al. 2020)
	Help to explore new technologies for managing especially chronic diseases including telemedicine and other approaches to reduce reliance on face-to-face clinic attendance especially during pandemics

NB, CVD, coronary vascular disease; LMICs, lower- and middle-income countries; NCDs, non-communicable diseases; PPE, personal protective equipment; T2DM, type 2 diabetes.

## Discussion

We believe this is the first study undertaken across Africa, as well as a number of Asian countries, to rapidly assess the impact of information, misinformation, and advice regarding pertinent technologies to prevent and treat COVID-19, including hydroxychloroquine with or without azithromycin, on subsequent utilisation, shortages and prices in the first few months following the pandemic. Pertinent medicines included antimalarials (hydroxychloroquine), antibiotics including azithromycin, analgesics as well as vitamins and immune boosters.

There were increases in the utilisation of antimalarials (hydroxychloroquine) across most African countries with the exception of Namibia, with more modest increases in Kenya and Malawi vs. for instance Nigeria. This may reflect greater control on the prescribing and dispensing of antimalarial treatments in these countries; however, further research is need before any definitive statements can be made. There was also increased utilisation of antimalarials in Bangladesh and Pakistan but not in Vietnam (Table 3). This may again reflect greater controls on the dispensing of antimalarials in Vietnam, although further research is needed to confirm this (Godman et al., 2020a).

There were also increases in the utilisation of antibiotics across most of the studied African countries as well as Bangladesh and Pakistan although typically more modest compared with changes in the utilisation of antimalarials, with an actual reduction in utilisation in Vietnam (Table 3). This may reflect a more conscious effort not to dispense antibiotics for suspected viral infections in Malawi, Namibia, and Vietnam; however, further research is needed before any definitive statements can be made. Increases in the utilisation of antimicrobials across countries is a concern as this will increase resistance rates (Llor and Bjerrum, 2014; Wojkowska-Mach et al., 2018; Wu et al., 2018; Godman et al., 2020b). Multiple strategies including educational strategies and antimicrobial stewardship programmes are typically needed among all key stakeholders in ambulatory care to reduce inappropriate prescribing and dispensing of antimicrobials, building on successful activities across lower- and middle-income countries (LMICs) (Bojanic et al., 2018; Mukokinya et al., 2018; Bishop et al., 2019; Jacobs et al., 2019; Godman et al., 2020b; Ogunleye et al., 2020). However, fining pharmacists for breaking current legislation may be difficult to enforce especially in rural areas of Africa where community pharmacists are typically the principal healthcare professional available and where there are no physician fees to contend with in the absence of universal healthcare (Kalungia and Godman, 2019; Alrasheedy et al., 2020). Other strategies may though be needed to reduce inappropriate dispensing of antimicrobials. These include greater education in pharmacy school to address information gaps regarding antimicrobials and antimicrobial resistance (AMR), developing and implementing national guidelines for the management of infectious diseases typically seen in ambulatory care acknowledging the WHO AWaRe list of antibiotics, as well as monitoring community pharmacists’ activities through for instance mobile technologies (Markovic-Pekovic et al., 2017; Hoxha et al., 2018; Kalungia and Godman, 2019; Saleem et al., 2019; Saleem et al., 2020). Community pharmacists should be mindful though that it can be difficult in practice to differentiate respiratory tract infections from COVID-19 among patients presenting with coughs and fever (Ongole et al., 2020), and they should encourage testing and referrals where there are concerns.

Encouragingly, there was increased use of vitamins/immune boosters across all the studied countries, with appreciable increases in some, mirroring the situation in Bangladesh, Pakistan and Vietnam (Table 3). Differences in utilisation rates between the countries may simply reflect different prevalence rates for COVID-19. However, more research is needed before any definitive statements can be made. There was also increased utilisation of analgesics among the studied countries; however, typically to a lesser degree than seen with vitamins/immune boosters. Again, differences may reflect different prevalence rates and other factors across countries. Encouragingly as well, there were appreciable increases in the utilisation of PPE where recorded (Table 3). This included Malawi where increases of up to two-fold were seen where recorded.

There was also appreciable increases in the prices for antimalarials (hydroxychloroquine) during the study period across a number of the studied countries including Bangladesh and Nigeria, with the latter building on earlier observations (Busari and Adebayo, 2020; Godman et al., 2020a; Haque et al., 2020a); however, to a lesser extent in Namibia and Pakistan (Table 4). This may well reflect some of the shortages seen in these countries (Table 5); however, further research is needed. We believe the limited price increases seen in Pakistan reflects greater price controls for medicines in the country than seen in a number of other countries (Lee et al., 2017; Godman et al., 2020a). There was a similar situation for antibiotics (principally azithromycin) (Tables 4 and 5) as well as for vitamins/immune boosters. Nigeria recorded the largest price increase among the African and Asian countries for antibiotics and vitamins (Table 4) potentially reflecting the shortages seen with vitamins but not necessarily with antibiotics (Table 5). Further studies though are needed before any definitive statements can be made.

Encouragingly as well, there were limited price increases for analgesics across the studied countries apart from Zambia, perhaps reflecting more limited increases in utilisation (Table 3). Where recorded, there were also price increases for PPE across the countries, again potentially reflecting appreciable increases in utilisation and shortages during the study period (Tables 3 and 5). However, again further research is needed before we can say anything with certainty. In Malawi, there were also price increase for PPE up to 50% to double in the majority of situations where this was recorded.

Lastly, it was also encouraging to see limited changes in the utilisation of herbal medicines among the studied countries (Table 3), with typically limited shortages apart from Ghana and Namibia. This is important given, as mentioned, that the inappropriate use of medicinal plants to prevent and treat COVID-19 can cause more harm than good (Ekor, 2014; Liwa et al., 2014; Nkeck et al., 2020; Nordling, 2020; Yang, 2020), and should be avoided until more information becomes available.

Potential ways forward to improve prevention and management of patients with COVID-19 among key stakeholder groups, and for future pandemics, are contained in Table 9. This includes potential ways to address concerns with shortages of medicines and PPE, including greater pro-activity, and suggesting possible alternatives with care, as well as increasing local production. These build on the suggestions among the pharmacists in Namibia (Table 8). We are already seeing activities within African countries, as well as consortium forming across Africa, to address current shortages with medicines and equipment including greater local production, and this is likely to grow, building on innovations with the production of ventilators across a number of African countries (Afriyie et al., 2020; EAC, 2020; Bissada, 2020; Kaine and Nwokik, 2020; Ogunleye et al., 2020; South African Government, 2020). However, there needs to be care with the quality of locally produced medicines given some concerns including the quality of generics (Fadare et al., 2016; Khan et al., 2016).

Suggestions outlined in Table 9 also include potential ways forward with community pharmacists, patient organisations, and others, to address the unintended consequences of COVID-19, especially if lockdown and other activities continue to adversely affect immunisation and prevention programmes for infectious diseases such as malaria. In addition, increase morbidity associated with NCDs such as CVD and T2DM if medicines are not being taken correctly. We will be following this up in the future.

We are aware of a number of limitations with this study. These include the fact that we only approached a limited number of pharmacists among the studied countries with the exception of Namibia as this was principally a pilot study in these countries to gain a rapid assessment of the current situation given concerns with shortages, price rises and deaths with some of the recommended medicines and strategies. In addition, we were unable to obtain exact details on changes in the utilisation and prices of pertinent medicines and PPEs from all the pharmacists visited due to issues of confidentiality, culture with lack of payment, available time with them and having the relevant information readily to hand when visited. Consequently, we could only collect impressions regarding any changes among a number of the visited pharmacists, which will be subject to bias. We were also unable to undertake any time series analysis or assess issues such as seasonality as we were primarily interested in changes post the pandemic; however, concerns with seasonality appear less of an issue among the African countries for the reasons stated. Despite these limitations, we believe our findings from this rapid study do provide direction for future research as well as address some of the concerns regarding the pandemic especially the impact of misinformation and shortages for patient care in this and other priority diseases.

## Conclusion

We have seen increases in the utilisation and prices for both antimalarials, principally hydroxychloroquine, and antibiotics, principally azithromycin, among the studied African and Asian countries arising from the COVID-19 pandemic, with considerable increases in some of the countries. These increases in antimicrobial utilisation need addressing where there are concerns through educational and other activities in order to prevent future rises in AMR. Community pharmacies, patient organisations, and others can play a key role in this respect as well as reduce the impact of any misinformation given the consequences experienced among countries.

Community pharmacists, patient organisations, and others, can also help address the unintended consequences from lockdown activities. These include addressing potential increases in infectious diseases from reduced immunisation and prevention programmes as well as helping to counteract greater morbidity from NCDs through addressing concerns with medicine availability and consumption, and we will be monitoring this in the future. Encouragingly, there was increased use of vitamins/immune boosters and PPE among the studied countries, which is likely to continue. However, this remains to be shown. The apparent considerable price rises seen for medicines and PPE in countries with existing high co-payment levels is a concern as this will impact on available resources for treating other priority infectious and non-infectious diseases. This urgently needs addressing, with community pharmacists again potentially playing an appreciable role alongside the Government.

## Data Availability

The raw data supporting the conclusions of this article will be made available by the authors, without undue reservation.
