# Supplementary material for: Rapid Assessment of the Potential Paucity and Price Increases for Suggested Medicines and Protection Equipment for COVID-19 Across Developing Countries With a Particular Focus on Africa and the Implications
Source: Front Pharmacol. 2021 Jan 14;11:588106. doi: 10.3389/fphar.2020.588106 (PMC7898676; doi:10.3389/fphar.2020.588106)
Supplement: Supplementary file 1 [file datasheet1.pdf]

Appendix 1A – Questionnaire used in Namibia

Serial number: ______________________________

101. Location of the pharmacy/dispensary?

- **Region: ______________________________**
- **Setting:** (1)☐ Rural (2) ☐ semi urban (3) ☐ urban
- **Facility type:** (1) ☐ **Private** (2) ☐ **Public**

102. Was the pharmacy prepared for this pandemic?

1-Yes 2- No

If yes, how?

1- Bulk ordering of medicines, sanitizers/disinfectants and PPE
2- Made arrangements for enough staff to be available

103. What key measures/interventions has the pharmacy put in place during this pandemic to curb the spread of COVID-19 in the community?

- Sanitization/disinfection/handwashing
- Social distancing protocols
- Personal protective equipment
- Temperature screening before entry
- Name register
- Multi-month dispensing
- Creating awareness

104. Itemize suggestions on the role (current and new) of pharmacist/pharmaceutical technicians/pharmacist assistant during this pandemic

- Educating and providing information on Covid-19 to the public
- Advising on treatment plan as well as providing the medicines
- Screening of patients that present to pharmacy and referring them for further testing if necessary
- Supply PPE and sanitizers
- Stock control

105. What were the main challenges experienced by the pharmacy during the pandemic?

- Inadequate stock of PPE
- Medicine stock-outs/ low stock
- Shortage of sanitizers/disinfectants
- Inadequate space in pharmacy for staff to maintain social distancing
- Increased workload due to initial panic buying and increased demand of items
- Clients/staff not adhering to regulations
- Increments in medicine prices hence impact on finances

106. What <u>INCREASE</u> in <u>SALES/DISPENSING/DEMAND</u> have you noticed since the beginning of March on the following items? (Give examples of the commonest types and estimate the percentage change if any)

	Tick for an increase **(✓)**	Increased by (%)	Specific Example
a) Sanitizers/Disinfectants			
b) Antimalarials			
c) Antibiotics			
d) Analgesics			
e) Herbal medications			
f) Multivitamins			
g) Immune boosters			
h) Cold and Flu medicines			
i) Contraceptives			
j) Other: ----------------------------			

107. What <u>INCREASES</u> in medicine <u>BUYING</u> <u>PRICE</u> (increase/decrease) have you noticed since the beginning of March on the following medicines? (estimate the percentage change in price)

	Tick for an increase **(✓)**	Increased by (%)	Specific Example
a) Sanitizers/Disinfectants			
b) Antimalarials			
c) Antibiotics			
d) Analgesics			
e) Herbal medications			
f) Multivitamins			
g) Immune boosters			
h) Cold and Flu medicines			
i) Contraceptives			
j) Other: ----------------------------			

108. What SHORTAGE/NON-AVAILABILITY of the following items have you noticed since the beginning of March? (Estimate stock out period in weeks/months if any)

	Tick for stockout **(✓)**	Estimated stockout periods in **Weeks/months**	Specific Example
a) Sanitizers/Disinfectants			
b) Antimalarials			
c) Antibiotics			
d) Analgesics			
e) Herbal medications			
f) Multivitamins			
g) Immune boosters			
h) Cold and Flu medicines			
i) Contraceptives			
j) Other: ----------------------------			

109. Which medicines/supplies in Namibia have experienced influx of fake/substandard/ spurious forms during this pandemic (if any)?

	Tick where applicable **(✓)**
a) Sanitizers/Disinfectants	
b) Antimalarials	
c) Antibiotics	
d) Analgesics	
e) Herbal medications	
f) Multivitamins	
g) Immune boosters	
h) Cold and Flu medicines	
i) Contraceptives	
j) Other: ----------------------------	

Thank you

References
